# How plausible is a subcortical account of rapid visual recognition?

**DOI:** 10.3389/fnhum.2013.00039

**Published:** 2013-02-27

**Authors:** Maxime Cauchoix, Sébastien M. Crouzet

**Affiliations:** ^1^Centre de Recherche Cerveau et Cognition, Université Paul Sabatier, Université de ToulouseToulouse, France; ^2^Faculté de Médecine de Purpan, CNRS, UMR 5549Toulouse, France; ^3^Department of Cognitive, Linguistic and Psychological Science, Brown UniversityProvidence, RI, USA; ^4^Institute of Medical Psychology, Charité University MedicineBerlin, Germany

Primates recognize objects in natural visual scenes with great rapidity. The ventral visual cortex is usually assumed to play a major role in this ability (“high-road”). However, the “low-road” alternative frequently proposed is that the visual cortex is bypassed by a rapid subcortical route to the amygdala, especially in the case of biologically relevant and emotional stimuli. This paper highlights the lack of evidence from psychophysics and computational models to support this “low-road” alternative. Most importantly, the timing of neural responses invites a serious reconsideration of the low-road role in rapid processing of visual objects.

## The speed of sight

The rapid and accurate processing of complex visual scenes has been demonstrated by Thorpe and colleagues using the rapid visual categorization protocol (Thorpe et al., 1996), in which participants reported the presence of animals in natural scenes as soon as 250 ms after image onset. This result sets strong time constraints on the neural mechanisms underlying object categorization. Diagnostic category information might actually be available even earlier, since selective eye movement responses can be produced only 100–120 ms after stimulus onset (Kirchner and Thorpe, 2006; Crouzet et al., 2010). What neural mechanisms could account for such rapid vision?

## The cortical “high-road”

A widely held view is that object recognition results from the interplay of hierarchically organized areas along the ventral visual stream (Dicarlo et al., 2012) from the primary visual cortex (V1) through extrastriate visual areas (V2 and V4), to the inferotemporal cortex (IT) where high-level visual representations are encoded. To reconcile this view with the short behavioral latencies observed in rapid categorization tasks, several authors have suggested that a pure feedforward sweep of activity through the ventral stream might be sufficient to perform core object recognition (Thorpe et al., 1996; Serre et al., 2007b).

## The subcortical “low-road”

On the other hand, a subcortical shortcut—the so-called “low-road”—might seem to be a plausible alternative. This hypothesis finds its origin in the rapid amygdala responses reported by (LeDoux, 1996) during auditory fear conditioning. In a series of experiments in rodents, he delineated a quick route that bypasses the cortex by directly reaching the amygdala via the thalamus. Such a subcortical shortcut would, in specific cases such as threatening situations, enable the rapid initiation of appropriate defense responses even before the sensory cortices become involved. Furthermore, since the amygdala has been linked to emotion recognition (particularly fear) in humans (Adolphs et al., 1994), this alternative pathway was proposed as an explanation for rapid, automatic, and unconscious reactions among humans and monkeys to biologically relevant visual stimuli (Öhman and Mineka, 2001; Johnson, 2005; Öhman, 2005; Vuilleumier, 2005; Tamietto et al., 2009; Tamietto and de Gelder, 2010; de Gelder et al., 2011).

Here we argue that there is no convincing evidence in support of the “low-road” theory when extended to rapid visual object processing in primates. To preface our arguments, first, real-world object categorization requires computational properties that have not yet been found in a subcortical pathway (see “Real-world Recognition Requires Selectivity and Invariance”). Second, among the characteristics attributed to the “low-road,” we argue that genuine rapidity has not yet been demonstrated appropriately (see “What is Rapid Visual Processing?”). Finally, we will demonstrate how the low-road hypothesis is at odds with neural latencies reported in the amygdala and the visual cortex (see “Ventral Stream Visual Cortex is Activated Before the Amygdala”). Altogether, these arguments point to an earlier role for cortical areas and suggest a serious reconsideration of the role of the “low-road” in rapid vision.

### Real-world recognition requires selectivity and invariance

To support recognition, a neural system needs to reach a high level of selectivity while dealing with the inherent variability of sensory input. This balance between selectivity and invariance is a hallmark feature of visual recognition in primates, and remains a challenge for computer vision.

In macaque monkeys, selective neural responses to complex objects are typically found in the IT (Dicarlo et al., 2012). These neuronal responses are also tolerant to changes in retinal position, scale, or pose of the object (Hung et al., 2005). Studies using intracranial recordings in human epileptic patients have also shown that neural responses from the visual cortex provide a categorical signal tolerant to changes in scale and position (Liu et al., 2009).

Driven by results from electrophysiology, a plausible model of how selectivity and invariance could be built through the ventral stream has emerged. It is based on two successive operations, template-matching and non-linear pooling, repeated at each stage of the ventral hierarchy (Serre et al., 2007b). Such hierarchical models have been shown to accurately mimic primate rapid categorization performance (Serre et al., 2007b; Crouzet and Serre, 2011) and neural responses of the visual ventral stream (Serre et al., 2007a).

Among subcortical structures, human single-unit studies showed that the amygdala contains neurons selective to categories or objects such as animals, famous faces, or places (Kreiman et al., 2000; Quiroga et al., 2005; Mormann et al., 2011). Interestingly, these neurons are highly invariant since they respond to various pictures of their preferential objects, but also to their written or spoken names. However, there is currently no model of how this high level of both selectivity and invariance could be built from a direct subcortical route. A more reasonable assumption would thus be that it gets its input from high-level areas of the ventral stream, rather than from the thalamus (shortcut “low-road” route).

### What is rapid visual processing?

Numerous studies investigated affective stimulus processing with short image presentation and masking protocols to show that emotions such as fear can be processed unconsciously and “rapidly” (Bar et al., 2006; Öhman et al., 2007; Adolphs, 2008). While there is no doubt that masking is a powerful experimental tool to reveal unconscious sensory processing, it does not provide information on the genuine rapidity of visual processing. In backward masking protocols, the stimulus onset asynchrony (SOA, time interval between target and mask onset) is a measure of the visual uptake time (or temporal resolution), not of the time required for complete visual processing. In other words, even in a perfect pipeline model of the visual system, the mask interference would only give information about the time spent at each stage, and not about the cumulative time for all stages (Vanrullen, 2011). For example, the fact that fear information can be extracted from faces masked after an SOA of 39 ms (Bar et al., 2006) informs us about the minimal visual uptake time necessary for fear processing but does not say anything about the time at which fear information is available to trigger behavioral responses.

The speed of processing for object or scene categorization has been extensively studied using rapid categorization protocols. Using minimal reaction time measurements (the time at which correct responses start to significantly outnumber incorrect ones) it has been shown that humans can categorize images as containing an animal in only 250 ms (Thorpe et al., 1996), while monkeys can perform the same task by 180 ms (Fabre-Thorpe et al., 1998). Even faster, reliable saccades toward faces and animals can be triggered as soon as 100–120 ms after image onset (Kirchner and Thorpe, 2006; Crouzet et al., 2010). As far as we know, there is no evidence for faster processing of emotional stimuli, as would be predicted by the “low-road” hypothesis.

### Ventral stream visual cortex is activated before the amygdala

Most of the studies on humans investigating the role of the amygdala in visual processing used fMRI and PET scans (Morris et al., 1999; Whalen et al., 2004; see Pessoa and Adolphs, 2010 and Vuilleumier, 2005 for reviews). These two techniques, because of their poor temporal resolution, do not allow conclusions about the temporal dynamics of stimulus processing. Despite this limitation, it was assumed that amygdala responses to emotional stimuli, notably to fear-inducing stimuli, were based on a rapid low-road activation (Öhman and Mineka, 2001; Vuilleumier, 2005).

A review of electrophysiological studies reporting neural latencies suggests a clearly different picture. Many studies investigating the properties of IT cells have reported selective responses to shapes, faces or object categories occurring as soon as 70–100 ms after stimulus onset (Perrett et al., 1982; Li et al., 1993; Tovee et al., 1994; Liu and Richmond, 2000; Hung et al., 2005; see Mormann et al., 2008 for a review). Similarly, in human epileptic patients, IFP recorded from the occipito-temporal cortex were object category selective as early as 100 ms after stimulus onset (Liu et al., 2009). These category selective latencies are compatible with the rapid behavioral responses observed in natural scene categorization tasks (Thorpe et al., 1996; Fabre-Thorpe et al., 1998; Kirchner and Thorpe, 2006; Girard et al., 2008). Furthermore, this early ventral stream activity has been shown to be causally linked with behavioral responses in monkeys (Afraz et al., 2006) and humans (Pitcher et al., 2007; Sadeh et al., 2011).

On the other hand, selective responses to visual features or objects in monkeys' amygdala tend to have a greater time-lapse (Gothard et al., 2007). One single-unit study (Leonard et al., 1985) compared the two pathways directly (on the same monkeys) and showed that neurons in the STS (superior temporal sulcus, top of the ventral stream) had latencies (90–140 ms) that systematically preceded those from the amygdala (110–200 ms). In humans, two intracranial recording studies have tested the existence of rapid amygdala responses to emotional stimuli. But the earliest responses were reported at 200 ms (Krolak-Salmon et al., 2004) and 250–500 ms (Rutishauser et al., 2011), which is much slower than the fast occipito-temporal selectivity reported for objects categories (Liu et al., 2009). Moreover, the amygdala responses observed for emotional stimuli were not occurring earlier than what is generally reported for object categories (Mormann et al., 2008). Among the medial temporal lobe structures (i.e., perirhinal cortex, entorhinal cortex, hippocampus, and amygdala), the amygdala is actually the one with the slowest visual responses (average latencies of 271 ms in the perirhinal cortex for example). The pattern of neural latencies observed in both human and monkeys thus clearly vouches for a cortical “high-road” precedence.

## Conclusion

In this paper we questioned the hypothesis that a subcortical low-road could account for the speed of sight. Several observations from psychophysics, computational modeling, and electrophysiology strongly suggest that the low-road account is mostly incompatible with the characteristics of rapid visual categorization. On the contrary, a large collection of evidence confirms that the cortical high-road, through the visual ventral stream, can accomplish a rapid, selective, and invariant analysis of the scene. The latency of neural visual activation and response characteristics in the amygdala clearly suggest that its involvement in visual processing is downstream of the ventral visual cortex, after core object recognition has been performed.

Thus, contrary to what is commonly acknowledged, rapid, and automatic processing of visual objects is likely to be under cortical-dependence while subcortical structures would be involved in slower (probably higher-level) processing. This conclusion conforms with recent results and reviews pointing out the unexpected role of subcortical structure in high cognitive functions (Parvizi, 2009). Amygdala for instance is now thought to play a major role in the evaluation of the biological significance of stimuli (Pessoa and Adolphs, 2010) and the pulvinar, showing dense connection with many cortical areas, has recently been shown to play a role in regulating information transmission across the visual cortex (Saalmann et al., 2012).
